# Extract from a Report of Medical Cases at Fort Pitt, Illustrative of the Large Doses of Digitalis Which May Occasionally Be Given with Advantage

**Published:** 1824-06

**Authors:** John Davy


ART. V.—*Extract from a Report of Medical Cases at Fort Pitt, illustrative of the large Doses of Digitalis which may occasionally be given with advantage.* By JOHN DAVY, M.D. F.R.S.

IN a case which is still in the hospital, but convalescent, a very remarkable recovery has taken place. The patient contracted the disease in India, where it followed an attack of ague and dysentery. During the last twelve months he has been tapped nine times. Now he is in pretty good health; his abdomen is reduced nearly to its natural size, and is without fluctuation. This favourable change, I think, may justly be attributed to the medicine he has used, viz. digitalis in a bitter infusion, with some subcarbonate of soda, and a minute quantity of blue-pill. I commenced with five grains of the powder of the leaf, and gradually increased it to *one hundred and fifteen grains* daily. It is worthy of remark, that this medicine operated and produced the good effect almost insensibly. When at its maximum dose, the pulse was rather quicker than natural; it never affected the head or stomach, and never increased the quantity of urine. It may be supposed, perhaps, that the preparation was not good: this I cannot admit, for in some cases of pulmonic disease, in which it was given, it produced the common effects of the drug, in the usual dose.

The case of anasarca, which was very severe, was completely cured by pulv. digitalis, gradually increased to *one hundred grains* daily. It is remarkable that, whilst the patient was using this medicine, his pulse was not apparently affected; but, on the day following that on which he discontinued it, the pulse suddenly fell from between seventy and eighty, (which it usually was,) to forty-six, and became irregular: next day it recovered its natural beat.

The case of ascites was attended with cough, pain of the right side of the chest and right hypochondrium, and was of three years' duration. It was cured by an alterative course of blue-pill, conium, and rhubarb, followed by pulv. digitalis, gradually increased to *one drachm* daily, exhibited in bitter infusion.*

---

* These cases are so interesting, from the immense doses of digitalis exhibited, that we shall endeavour to procure them at full length, on some future occasion. —EDITORS.

